# Comparison of three nucleic acid-based tests for detecting *Anaplasma marginale* and *Anaplasma centrale* in cattle

**DOI:** 10.4102/ojvr.v84i1.1262

**Published:** 2017-01-23

**Authors:** Mamohale E. Chaisi, Janine R. Baxter, Paidashe Hove, Chimvwele N. Choopa, Marinda C. Oosthuizen, Kelly A. Brayton, Zamantungwa T.H. Khumalo, Awelani M. Mutshembele, Moses S. Mtshali, Nicola E. Collins

**Affiliations:** 1 Department of Veterinary Tropical Diseases, University of Pretoria, South Africa; 2 Department of Genetics, University of Pretoria, South Africa; 3 Biotechnology Platform, Agricultural Research Council, South Africa; 4 Department of Veterinary Services, Ministry of Agriculture and Livestock, Zambia; 5 Department of Veterinary Microbiology and Pathology, Washington State University, United States; 6 National Zoological Gardens, Pretoria, South Africa

## Abstract

Several nucleic acid-based assays have been developed for detecting *Anaplasma marginale* and *Anaplasma centrale* in vectors and hosts, making the choice of method to use in endemic areas difficult. We evaluated the ability of the reverse line blot (RLB) hybridisation assay, two nested polymerase chain reaction (nPCR) assays and a duplex real-time quantitative polymerase chain reaction (qPCR) assay to detect *A. marginale* and *A. centrale* infections in cattle (*n* = 66) in South Africa. The lowest detection limits for *A. marginale* plasmid DNA were 2500 copies by the RLB assay, 250 copies by the nPCR and qPCR assays and 2500, 250 and 25 copies of *A. centrale* plasmid DNA by the RLB, nPCR and qPCR assays respectively. The qPCR assay detected more *A. marginale-* and *A. centrale*-positive samples than the other assays, either as single or mixed infections. Although the results of the qPCR and nPCR tests were in agreement for the majority (38) of *A. marginale*-positive samples, 13 samples tested negative for *A. marginale* using nPCR but positive using qPCR. To explain this discrepancy, the target sequence region of the nPCR assay was evaluated by cloning and sequencing the *msp1β* gene from selected field samples. The results indicated sequence variation in the internal forward primer (AM100) area amongst the South African *A. marginale msp1β* sequences, resulting in false negatives. We propose the use of the duplex qPCR assay in future studies as it is more sensitive and offers the benefits of quantification and multiplex detection of both *Anaplasma* spp.

## Introduction

Bovine anaplasmosis is a tick-borne disease of cattle caused by the intra-erythrocytic rickettsia, *Anaplasma marginale* (Theiler 1910). The clinical manifestations of anaplasmosis include fever, progressive anaemia and icterus, and the disease has a case fatality rate of up to 36% (Losos 1986). Anaplasmosis is widely distributed around the world, and in South Africa, it is endemic in most of the cattle-farming areas (De Waal 2000; Marufu et al. 2010; Mtshali et al. 2007; Potgieter 1979; Stevens et al. 2007). Five tick species have been implicated in the transmission of *A. marginale* in South Africa: *Rhipicephalus decoloratus*, *Rhipicephalus microplus*, *Rhipicephalus evertsi evertsi*, *Rhipicephalus simus* and *Hyalomma marginatum rufipes* (Potgieter 1979; Potgieter & Van Rensburg 1980).

*Anaplasma marginale* subsp. *centrale*, commonly referred to as *Anaplasma centrale*, was first isolated in South Africa by Sir Arnold Theiler (Theiler 1911) who originally classified it as ‘*A. marginale* variety *centrale*’. It causes a milder form of anaplasmosis, and a live blood vaccine containing *A. centrale* is used to immunise cattle against *A. marginale* in many countries, including South Africa (De Waal 2000; Melendez et al. 2003; Potgieter & Van Rensburg 1983). However, this vaccine causes variable protection against *A. marginale* and might not be effective against antigenically diverse, highly virulent stocks of *A. marginale* (Bock & De Vos 2001). The vaccine strain can cause reactions in adult cattle of susceptible breeds (Bigalke 1980; Pipano 1976) and it has been reported to cause severe anaplasmosis in splenectomised adult cattle (Kuttler 1966; Pipano, Mayer & Frank 1985). More recently, a strain of *A. centrale* that is closely related to the vaccine strain was associated with a case of clinical disease in a bovine in Europe (Carelli et al. 2008).

The seroprevalence of *A. marginale* in South Africa is known to be high (Mtshali et al. 2007; Stevens et al. 2007) and a number of novel *A. marginale* strains have been identified by the analysis of *msp1α* genotypes (De la Fuente et al. 2007; Mtshali et al. 2007; Mutshembele et al. 2014). However, little work has been performed on the molecular detection of *A. marginale* and *A. centrale* in the field in South Africa. Infection by these organisms in endemic regions is usually low, asymptomatic and contribute to transmission by vectors. As these low infections can only be effectively detected using molecular methods (Hofmann et al. 2015; Schotthoefer et al. 2013; Strik et al. 2007), various assays have been developed to detect *A. marginale* and *A. centrale* DNA in vectors and hosts in different parts of the world. These include the reverse line blot (RLB) hybridisation assay (Bekker et al. 2002), restriction fragment length polymorphism (RFLP) assays (Noaman & Shayan 2010), nested polymerase chain reaction (nPCR) assays (Decaro et al. 2008; Molad et al. 2006) and quantitative real-time polymerase chain reaction (qPCR) assays (Carelli et al. 2007; Decaro et al. 2008; Futse et al. 2003; Picoloto et al. 2010; Reinbold et al. 2010; Ueti et al. 2007). Most of these assays have only been used to follow the organisms in experimentally infected cattle; however, the nPCR assay designed by Molad et al. (2006) and the qPCR tests developed by Carelli et al. (2007) and Decaro et al. (2008) have been used to detect *A. marginale* and *A. centrale* in field samples in Israel and Italy.

The availability of all these molecular diagnostic assays of different sensitivities and cost makes the choice of an appropriate test for epidemiological studies difficult (Bacanelli, Ramos & Araujo 2014). It is also important to assess the suitability of these assays in the detection of local *A. marginale* and *A. centrale* strains, as many different strains of *A. marginale* have been reported in South Africa (Mtshali et al. 2007; Mutshembele et al. 2014) and elsewhere around the world (Almazan et al. 2008; Cabezas-Cruz et al. 2013; De la Fuente et al. 2001, 2007; Pohl et al. 2013).

We evaluated the ability of three different techniques, the RLB hybridisation assay (Bekker et al. 2002), nPCR assays (Decaro et al. 2008; Molad et al. 2006) and a duplex qPCR assay (Decaro et al. 2008), in detecting *A. marginale* and *A. centrale* infections in cattle in South Africa. To explain discrepancies between the nPCR and qPCR assay results in the detection of *A. marginale*, the target sequence region of the nPCR assay was evaluated by cloning and sequencing the *msp1β* gene from selected *A. marginale*-positive field samples.

## Methods

### Sample collection and DNA Extraction

A total of 66 blood samples originating from cattle in Mpumalanga (*N* = 42), Western Cape (*N* = 13) and KwaZulu-Natal (*N* = 11) provinces in South Africa were included in the study. The samples were either obtained as frozen blood samples (obtained from the National Zoological Gardens, Pretoria) or collected as fresh blood samples from cattle in the Mnisi Community area, Bushbuckridge, Mpumalanga province, South Africa. Fresh blood samples were collected in 9-mL Vacutainer^®^ EDTA tubes from the caudal vein of cattle that were at least 1-year old in accordance with the animal ethics code of the University of Pretoria. Genomic DNA was extracted from the blood samples using a QIAamp DNA Blood Mini Kit (Qiagen, USA), according to the manufacturer’s instructions.

### Detection of *Anaplasma marginale* and *Anaplasma centrale*

The samples were analysed for the presence of *A. marginale* and *A. centrale* using three PCR-based methods.

### Reverse line blot hybridisation assay

Primers Ehr-F and Ehr-R (Table 1) were used to amplify the V1 hypervariable region of the 16S rRNA gene of *Anaplasma* and *Ehrlichia* species present in the samples. The PCR was performed in a 25-µL reaction mixture containing 1X Platinum Quantitative PCR Supermix UDG (Invitrogen), 3 mM MgCl_2_, 200 µM dNTPs, 0.2 µM of each primer and 2.5 µL of template DNA (approximately 200 ng). A touchdown thermal cycling programme was used as previously described (Nijhof et al. 2005). PCR products were subjected to RLB hybridisation as described by Nijhof et al. (2005) using the genus- and species-specific oligonucleotide probes reported in Bekker et al. (2002).

**TABLE 1:** Oligonucleotide primers and probes used in this study for the detection of *Anaplasma marginale* and *Anaplasma centrale.*

Assay	Target gene	Oligonucleotide name	Sequence (5’–3’)	Amplicon size (bp)	Reference
**Reverse line blot**					
Amplification primers	16S rRNA	Ehr-F	GGAATTCAGAGTTGGATCMTGGYTCAG	498	Bekker et al. (2002)
		Ehr-R	Biotin-CGGGATCCCGAGTTTGCCGGGACTTYTTCT	-	-
*Anaplasma marginale*	-	Am probe	GACCGTATACGCAGCTTG	-	-
*Anaplasma centrale*	-	Ac probe	TCGAACGGACCATACGC	-	-
**Duplex quantitative polymerase chain reaction**					
*Anaplasma marginale*	*msp1β*	AM-For	TTGGCAAGGCAGCAGCTT	95	Carelli et al. (2007)
		AM-Rev	TTCCGCGAGCATGTGCAT	-	-
		AM-Pb	6FAM – TCGGTCTAACATCTCCAGGCTTTCAT – BHQ1	-	-
*Anaplasma centrale*	*groEL*	AC-For	CTATACACGCTTGCATCTC	77	Decaro et al. (2008)
		AC-Rev	CGCTTTATGATGTTGATGC	-	-
		AC-Pb	LC610 – ATCATCATTCTTCCCCTTTACCTCGT – BHQ2	-	-
**Nested polymerase chain reaction**					
*Anaplasma marginale*	*msp1β*	AM456	CCATCTCGGCCGTATTCCAGCGCA (primary PCR)	732	Molad et al. (2006)
		AM1164	CTGCCTTCGCGTCGATTGCTGTGC	-	-
		AM100	CAGAGCATTGACGCACTACC (secondary PCR)	246	-
		AM101	TTCCAGACCTTCCCTAACTA	-	-
*Anaplasma centrale*	*msp2*	AC1826	TTGTGGCTCTAGTCCCCCGGGGAG (primary PCR)	566	Molad et al. (2006)
		AC2367	AGACAAAGAACCCGGCGTAGCAGCTC	-	-
		CIS1925	TTCTTGAGCAGGGGGATACC (secondary PCR)	252	-
		CIS2157	AGACCCGGCGGA AATACCAT	-	-
**Conventional polymerase chain reaction (for cloning and sequencing)**					
*Anaplasma marginale*	*msp1β*	Am.F	ATGACAGAAGACGACAAGCAAC	1900	Molad et al. (2006)
		Am.R	AGTAACAATTGCTTGGTCGT	-	-
*Anaplasma centrale*	*groEL*	groEL-ACF	TCTTCTTCTGACTACGACAAGGAAAAACTG	488	Decaro et al. (2008)
		groEL-ACR	GTCATGAATACAGCTGCRAGTGACACAGCC	-	-
*Anaplasma marginale* and *Anaplasma centrale*	16S rRNA	rD1	AGAGTTTGATCCTGGCTCAG	1500	Weisburg et al. (1991)
		rP2	ACGGCTACCTTGTTACGACTT	-	-

PCR, polymerase chain reaction.

### Duplex real-time quantitative polymerase chain reaction

The samples were analysed using the duplex qPCR assay reported by Decaro et al. (2008) for simultaneous detection of *A. marginale* (detecting the *msp1β* gene) and *A. centrale* (detecting the *groEL* gene), with minor modifications of the *A. centrale* probe to adapt it for use in the Lightcycler real-time PCR system. The 20 µL reaction mixture contained 4 µL of FastStart Taqman mix (Roche Diagnostics), 0.5 µL UDG, 0.6 µM of *A. marginale-*specific primers AM-For and AM-Rev (Table 1), 0.9 µM of *A. centrale*-specific primers AC-For and AC-Rev (Table 1), 0.2 µM of probes AM-Pb and AC-Pb (Table 1) and 2.5 µL of template DNA (approximately 200 ng). DNA extracted from the *A. centrale* vaccine strain purchased from Onderstepoort Biological Products (OBP) and sample 9410 obtained from Dr Helena Steyn, Onderstepoort Veterinary Institute (OVI), Pretoria, South Africa, were used as positive controls for *A. centrale*. Sample 9410 was confirmed to have *A. centrale* infection by amplification and sequence analysis of the *groEL, msp2* and 16S rRNA genes. Samples C14 and F48 (originating from bovines in the Mnisi Community area) were used as positive controls for *A. marginale*. These samples were confirmed to contain *A. marginale* infections by amplification and sequence analysis of the *msp1b* gene. Nuclease-free water was used as a negative control. Thermal cycling was performed in a LightCycler v2 (Roche Diagnostics, Mannheim, Germany). Thermal cycling conditions were UDG activation at 40 °C for 10 min, pre-incubation at 95 °C for 10 min, 40 cycles of denaturation at 95 °C for 1 min, annealing–extension at 60 °C for 1 min and a final cooling step at 40 °C for 30 s. The results were analysed using the Lightcycler Software version 4.0 (Roche Diagnostics, Mannheim, Germany). A positive result was indicated by a Cq value (quantification cycle, synonymous with the Cp, crossing point, value given by the Lightcycler instrument), the cycle at which fluorescence from amplification exceeds the background fluorescence. A lower Cq correlates with a higher starting concentration of target DNA in a sample. FAM fluorescence (530 nm) was generated in *A. marginale*-positive samples, and LC-610 signals (610 nm) were generated in *A. centrale*-positive samples.

### Nested polymerase chain reaction

Two nPCRs were used to detect *A. marginale* and *A. centrale* in the samples as previously described (Decaro et al. 2008; Molad et al. 2006). External primers AM456 and AM1164 and internal primers AM100 and AM101 (Table 1), specific for the *msp1β* gene of *A. marginale*, were used in the *A. marginale*-specific nPCR. External primers AC1826 and AC2367 and internal primers CIS1925 and CIS2157 (Table 1) were used to detect the *msp2* gene of *A. centrale*. The optimised PCRs were performed in a final volume of 25 μL, containing 1X DreamTaq Green PCR master mix (ThermoFisher Scientific, South Africa), yielding final concentrations of 2 mM MgCl_2_, 0.2 mM dNTPs, 1X DreamTaq™ reaction buffer and a proprietary amount of DreamTaq™ DNA polymerase. For both primary PCRs, approximately 200 ng of genomic DNA was used as template, and each external primer was added to a final concentration of 0.5 μM. The primary PCR thermal cycling conditions were 95 °C for 3 min, 35 cycles of 95 °C for 10 s, 62 °C for 30 s and 72 °C for 30 s, followed by a final extension at 72 °C for 7 min. The secondary PCR reaction mixes were prepared in the same way, except that each internal primer was added to a final concentration of 1 μM, and 1 μL of a 1:100 dilution of the primary PCR product was added as template. The secondary PCR thermal cycling conditions were the same as the primary PCR cycling protocol, except that the annealing temperature was at 66 °C (*A. marginale*) and 68 °C (*A. centrale*). The secondary PCR products were analysed by electrophoresis through a 2% agarose gel and stained with ethidium bromide.

### Specificity and sensitivity of the reverse line blot, nested polymerase chain reaction and quantitative polymerase chain reaction assays

The specificity of the RLB, nPCR and qPCR assays in detecting closely related species has previously been assessed (Bekker et al. 2002; Carelli et al. 2007; Decaro et al. 2008; Molad et al. 2006). We analysed DNA extracted from *Anaplasma* sp. Omatjenne, *Anaplasma phagocytophilum*, *Babesia bovis* and *Theileria parva* using the RLB, nPCR and qPCR assays.

In order to determine the sensitivities of the assays, the *msp1β* and 16S rRNA genes of *A. marginale* from sample F48, and the *groEL*, *msp2* and 16S rRNA genes of *A. centrale* from sample 9410 were amplified with gene-specific primers (Table 1) and cloned in the pJET vector (ThermoFisher Scientific, South Africa). Clones with the correct insert were sequenced at Inqaba Biotechnologies (South Africa) using vector primers. The sequences were assembled and aligned using the CLC Main Workbench 7 (http://www.clcbio.com). Plasmid DNA was extracted from clones F48a (*A. marginale msp1β* gene), F48d (*A. marginale* 16S rRNA gene), 9410c (*A. centrale groEL* gene), 9410g (*A. centrale* 16S rRNA gene) and 9410i (*A. centrale msp2* gene) using the High Pure Plasmid Isolation Kit (Roche Diagnostics, Mannheim, Germany). The concentrations of the plasmids were determined using the PowerWave XS2 Microplate Spectrophotometer (Biotek, USA), and the copy number (copies/μL) was calculated using the formula below (Ke et al. 2006):

$$\text{Copy number}=\frac{6\times 10^{23}(\text{copies/mol})\times \text{concentration }\left(g/L\right)}{\text{DNA length }\left(bp\right)\times 660\left(g/mol/bp\right)}$$ [Eqn 1]

The linear ranges of detection of the assays were evaluated by analysing 10-fold serial dilutions of plasmid DNA using the RLB, qPCR and nPCR with an input of 2.5 μL of each dilution of DNA. For the qPCR duplex assay, the dilutions were analysed in triplicate, and the means of the Cq values were plotted against the log concentrations to generate standard curves for absolute quantification of *A. marginale* and *A. centrale*. PCR efficiency (E) was calculated from the slope of the curve using the formula below (Bustin et al. 2009):

$$E=10^{1/\text{slope}}-1$$ [Eqn 2]

### Amplification, cloning and sequencing of the msp1*β* gene

To explain discrepancies between the nPCR and qPCR assay results in the detection of *A. marginale*, the target sequence region of the nPCR assay was evaluated by cloning and sequencing of the *msp1β* gene from selected *A. marginale*-positive field samples (C1, C14, C57, F48). Primers Am.F and Am.R or AM456 and AM1164 (Table 1) were used for the PCR. The 25 µL reaction mixture contained 1x DreamTaq Green PCR master mix (ThermoFisher Scientific, South Africa), 0.5 µM of each primer (Table 1) and 2.5 µL of template DNA. Purified PCR products were cloned into pGEM^**®**^-T (Promega, USA), and recombinant plasmids were sequenced at Inqaba Biotechnologies (South Africa). The sequences were assembled and analysed using the CLC Main Workbench 7 (http://www.clcbio.com). The identity of sequences obtained was determined by BLAST analysis (Altschul et al. 1990), using the *blastn* function.

### GenBank accession numbers

Sequences were submitted to GenBank under the following accession numbers: KU647713–KU647720 *(A. marginale msp1β* gene), KU598853 (*A. marginale* 16S rRNA gene), KU598854 (*A. centrale* 16S rRNA gene), KU647711 (*A. centrale groEL* gene) and KU647712 (*A. centrale msp2* gene).

### Statistical analysis

The data were analysed using the Statistical Package for the Social Sciences (SPSS) version 23.0 (IBM SPSS, 2014). The Fisher’s exact test was used to determine if the results of RLB, nPCR and qPCR assays in detecting *A. marginale* or *A. centrale* infections in cattle were significantly different. The level of agreement between the results of the three assays was evaluated using the Kappa score, at a 95% confidence interval (Viera & Garrett 2005).

## Results

### Specificity and sensitivity of the assays

Amplicons of approximately 500 bp were obtained from *A. marginale* clone F48d and *A. centrale* clone 9410g using the RLB PCR primers. Amplicons of 246 bp (from *A. marginale* clone F48a) and 252 bp (from *A. centrale* clone 9410i) were obtained using nPCR. As expected, qPCR products of approximately 95 bp and 77 bp were obtained from *A. marginale* clone F48a and *A. centrale* clone 9410c, respectively (results not shown). FAM fluorescence (530 nm) was generated from *A. marginale* clone F48a, and LC-610 (610 nm) signals were generated from *A. centrale* clone 9410c. The efficiency of the duplex qPCR was 104% and 101% (Figure 3) for *A. marginale* and *A. centrale*, respectively. No amplification was detected from the DNA of *Anaplasma* sp. Omatjenne, *A. phagocytophilum*, *B. bovis* and *T. parva* or from the negative (water) control by the RLB, nPCR or duplex qPCR assay.

**FIGURE 1:**
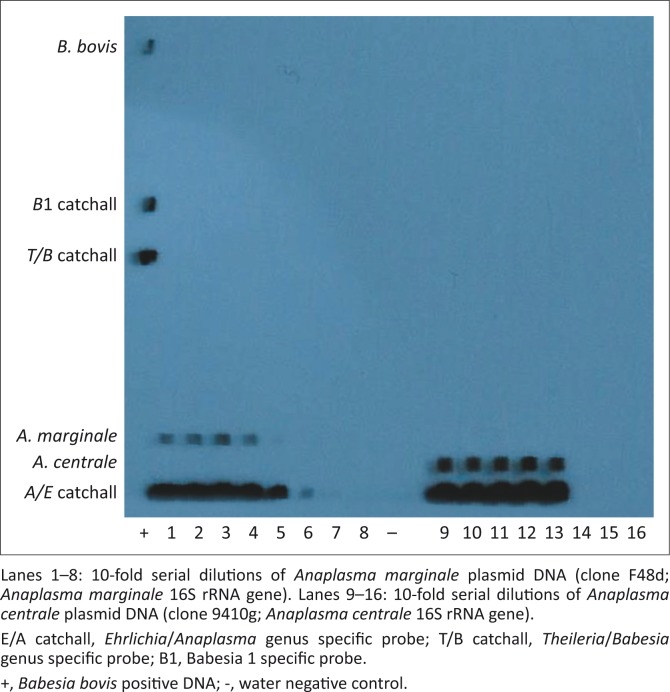
Detection of serial dilutions (2.5x10^7^ – 2.5x10^0^ copies) of plasmid DNA by the reverse line blot hybridisation assay.

**FIGURE 2:**
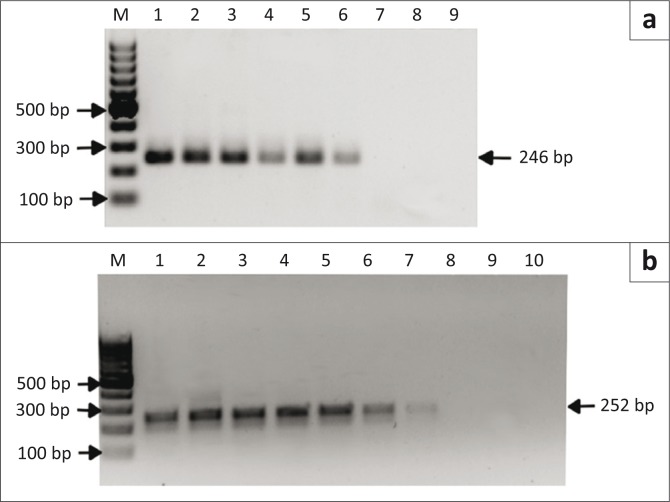
Detection of serial dilutions of plasmid DNA by nested polymerase chain reaction. (a) Lanes 1–8: 10-fold serial dilutions (2.5x10^7^ – 2.5x10^0^ copies) of *Anaplasma marginale* plasmid DNA (clone F48a; *msp1β* gene); lane 9: water negative control. (b) Lanes 1–9: 10-fold serial dilutions (2.5x10^8^ – 2.5x10^0^ copies) of *Anaplasma centrale* plasmid DNA (clone 9410i; *msp2* gene); lane 10: water negative control. M: 100 base pair marker; numbers on the left and right indicate molecular sizes in base pairs.

**FIGURE 3:**
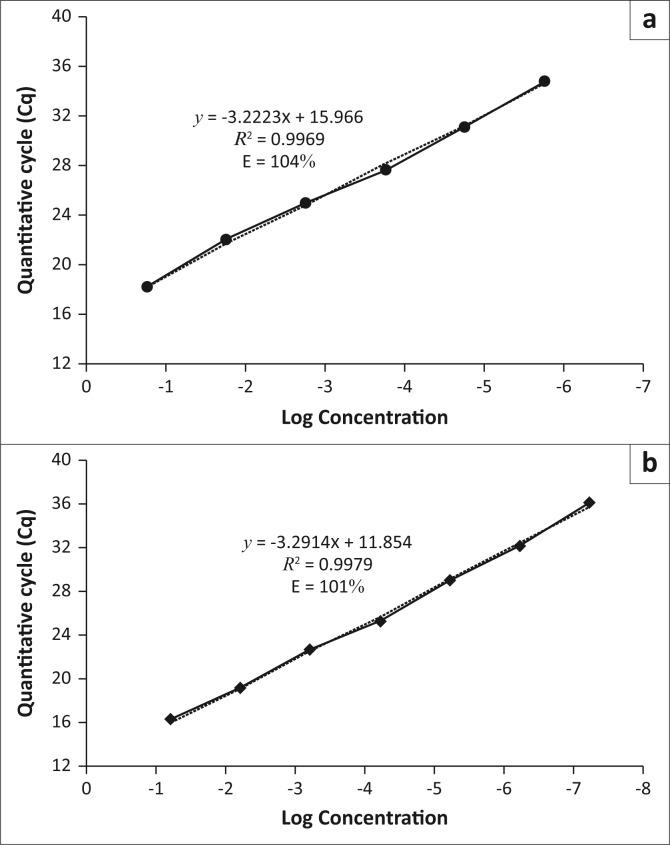
Detection of 10-fold serial dilutions of plasmid DNA by the duplex quantitative polymerase chain reaction assay. (a) *Anaplasma marginale* plasmid DNA (clone F48a; *msp1β* gene) 2.5x10^7^ – 2.5x10^2^ copies. (b) *Anaplasma centrale* plasmid DNA (clone 9410c; *groEL* gene) 2.5 x 10^7^ – 2.5 x 10^1^ copies.

Serial dilutions of each plasmid clone were prepared and tested using the RLB hybridisation assay (*A. marginale* clone F48d and *A. centrale* clone 9410g), nPCR (*A. marginale* clone F48a and *A. centrale* clone 9410i) and duplex qPCR (*A. marginale* clone F48a and *A. centrale* clone 9410c). The smallest amounts of *A. marginale* plasmid DNA that could be detected were 2500 copies per reaction for the RLB hybridisation assay (Figure 1) and 250 copies per reaction for the nPCR and qPCR assays (Figures 2a and 3a). *Anaplasma centrale* detection limits by the RLB, nPCR and qPCR assays were 2500, 250 and 25 copies per reaction respectively (Figures 1, 2b and 3b).

### Detection of *Anaplasma marginale* and *Anaplasma centrale* in field samples by the reverse line blot, nested polymerase chain reaction and quantitative polymerase chain reaction assays

**FIGURE 4:**
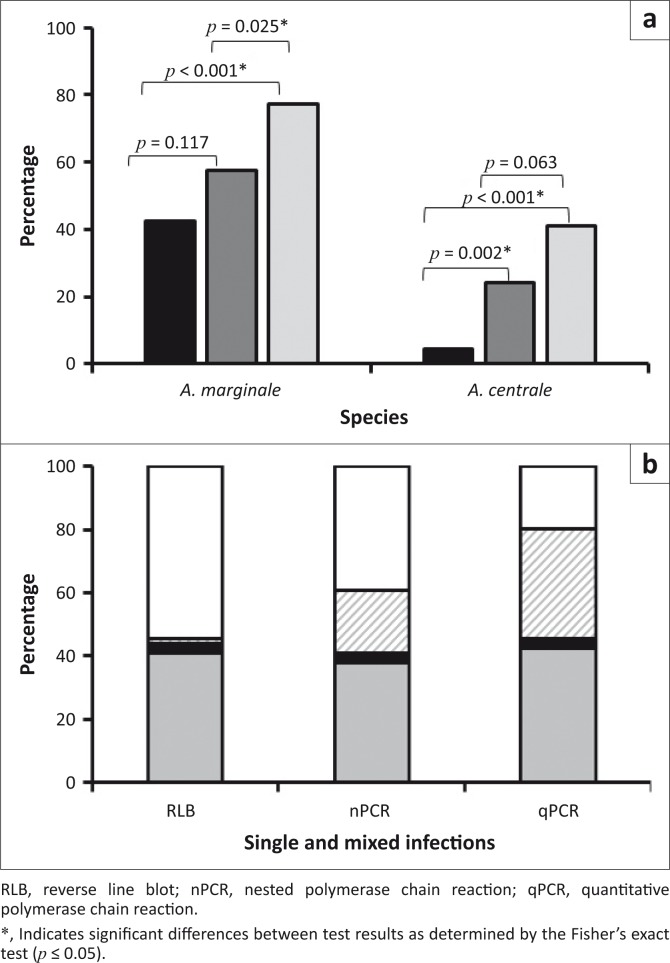
(a) Detection of *Anaplasma marginale* and *Anaplasma centrale* in South African cattle samples (*n* = 66) by the reverse line blot hybridisation assay (black), nested polymerase chain reaction (dark grey) and quantitative polymerase chain reaction (light grey). (b) Proportion of single and mixed infections in South African cattle samples as detected by the three assays. Single *Anaplasma marginale* infection (grey), single *Anaplasma centrale* infection (black), mixed *Anaplasma marginale* and *Anaplasma centrale* infections (hatched), no infection detected (white).

The qPCR assays detected more *A. marginale-* and *A. centrale*-positive samples than either the RLB or nPCR assays (Figure 4a), either as single or mixed infections (Figure 4b), although this difference was not statistically significant for *A. centrale* infections detected by the qPCR and nPCR (Figure 4a). The number of *A. marginale*-positive samples detected by qPCR was significantly different from the number of *A. marginale*-positive samples detected by RLB or nPCR (*p* ≤ 0.05). Both nPCR and qPCR detected significantly more *A. centrale*-positive samples than the RLB (*p* ≤ 0.05; Figure 4a). There was no significant difference between the number of *A. marginale* infections detected by the RLB and nPCR assays (Figure 4b).

The level of agreement between the results of the three assays was determined using Kappa scores (Table 2). For *A. marginale*, the agreement between the RLB and nPCR assays and between the RLB and the qPCR assay was fair, whereas the agreement between the nPCR and qPCR was moderate. For *A. centrale,* there was slight agreement between the results of the RLB and the nPCR assays, and between the RLB and qPCR assays. The agreement between the nPCR and the qPCR results was substantial (Table 2).

**TABLE 2:** Comparison of reverse line blot, nested polymerase chain reaction and quantitative polymerase chain reaction assays in the detection of *Anaplasma marginale* and *Anaplasma centrale* in cattle samples in South Africa.

Species	Reverse line blot			Nested polymerase chain reaction		
	+	-	Kappa value (95% CI)	+	-	Kappa value (95% CI)
***Anaplasma marginale* – nPCR**						
+	20	8	0.23^a^ (0.024–0.456)*	n/a	n/a	n/a
-	18	20		n/a	n/a	
***Anaplasma marginale –* qPCR**						
+	26	2	0.244^a^ (0.072–0.417)*	38	0	0.571^b^ (0.39–0.759)*
-	25	13		13	15	
***Anaplasma centrale* – nPCR**					
+	2	1	0.145^c^ (0.113–1.755)	n/a	n/a	n/a
-	14	49		n/a	n/a	
***Anaplasma centrale* – qPCR**					
+	3	0	0.129^c^ (0.00–0.282)*	16	0	0.632^d^ (0.434–0.807)*
-	24	39		11	39	

nPCR, nested polymerase chain reaction; qPCR, quantitative polymerase chain reaction.

a Fair agreement (0.21–0.40);

b moderate agreement (0.41–0.60);

c slight agreement (0.01–0.20);

d substantial agreement (0.61–0.80).

* *p* ≤ 0.05.

The nPCR and the qPCR assays had equivalent sensitivities in detecting *A. marginale* plasmid dilutions, and therefore, a substantial agreement between the tests was expected. However, the agreement was only moderate. Although the two tests were in agreement for the majority (38) of *A. marginale*-positive samples, 13 samples that tested positive by the qPCR assay tested negative by nPCR (Table 2). DNA smears were obtained in many of the *A. marginale msp1β* secondary PCR products from field samples, compared with clear bands obtained for *A. centrale groEL* secondary PCR products (results not shown).

**FIGURE 5:**
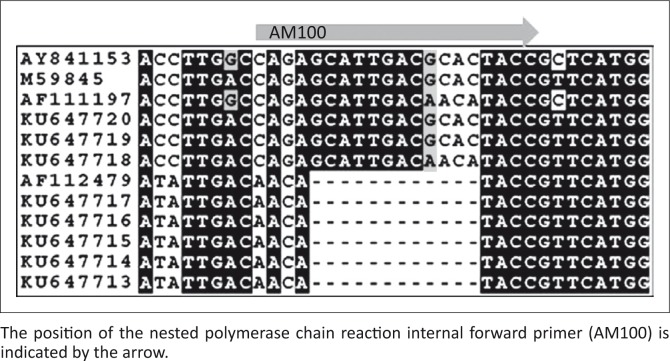
Alignment of South African *Anaplasma marginale msp1β* sequences generated in this study (KU647713–KU64747420) with published *Anaplasma marginale msp1β* sequences M59845 (Florida), AF111196 and AF111197 (South Idaho) and AF112479 (Havana).

To investigate the discrepancy in the detection of *A. marginale* by the nPCR and qPCR assays, the *msp1β* gene was amplified, cloned and sequenced from selected field samples that yielded both sharp and ‘smeary’ PCR products to determine whether the target sites of the *A. marginale* nPCR primers were conserved in the field samples examined. The sequence alignment indicated that the target sites of the external primers, AM456 and AM1164, and the internal reverse primer, AM101, were identical in all of the samples. However, the target site of the internal forward primer AM100 was not well conserved amongst the different *A. marginale msp1β* gene sequences from South Africa (Figure 5).

The nPCR and the qPCR tests were in agreement for 16 *A. centrale*-positive samples, but 11 samples tested positive for *A. centrale* using qPCR and negative using nPCR (Table 2). An attempt was made to amplify the *msp2* gene from these samples, but it was not possible to obtain visible PCR products because of very low rickettsemias, and therefore sequence data could not be obtained.

## Discussion

In epidemiological studies, data on the prevalence of parasitic infections is highly dependent on the sensitivity of the diagnostic assay used (Hofmann et al. 2015). We evaluated the ability of three published molecular assays in detecting *A. marginale* and *A. centrale* infections in blood samples from cattle in South Africa. The RLB (Bekker et al. 2002), nPCR (Decaro et al. 2008; Molad et al. 2006) and qPCR (Carelli et al. 2007; Decaro et al. 2008) assays have previously been shown to be specific for detecting these infections in tick vectors and hosts. In addition, our results indicated that the tests do not detect DNA from *Anaplasma* sp. Omatjenne, a species frequently encountered in South African field samples.

Our results indicate that the RLB assay is less sensitive than the nPCR and qPCR assays in detecting *A. marginale* and *A. centrale* infections in cattle under the conditions prevailing when the tests were performed in South Africa. The RLB assay is nevertheless a valuable screening tool for simultaneously detecting infections using species and genus specific (catchall) probes (Bekker et al. 2002; Gubbels et al. 1999). It has therefore been used extensively to reveal *Anaplasma*/*Erhlichia* and/or *Babesia*/*Theileria* infections in different hosts and vectors and in identifying novel species and variants of species in these genera (Bhoora et al. 2009; Bosman et al. 2010; Ceci et al. 2014; Chaisi et al. 2011; Mans et al. 2011; Nijhof et al. 2005; Oosthuizen et al. 2009). In samples that contain mixed infections, however, the use of a single primer pair to amplify all infections decreases the sensitivity of the assay because of competition for primers between the different templates. Organisms present at low infection levels could be masked by those with higher infection levels and could therefore be missed. Misdiagnosis of carrier animals has important implications for disease control as outbreaks may occur when such animals are introduced to naïve animals in the presence of tick vectors (Bilgic et al. 2013). In our study, the ‘catchall’ probe signal was very strong at the lowest detection limit of *A. marginale*, but the species-specific signal was very weak (Figure 1). Such low infections could easily be missed or regarded as ‘catchall’ signals only. Optimisation of the concentration of the *A. marginale* probe used in the RLB assay might help in overcoming this problem. The *A. centrale* species-signal was strong and remained so throughout the detection range of the assay (Figure 1).

The nPCR and duplex qPCR assays, which both detect the *msp1β* gene of *A. marginale* (Carelli et al. 2008; Molad et al. 2006), had the same detection limit (250 copies/reaction) in detecting *A. marginale* plasmid clones. However, the qPCR assay detected significantly more infections from field samples than the nPCR assay. In other studies, the nPCR assay was reported to be equally sensitive to the qPCR in detecting *A. marginale* infections (Carelli et al. 2007; Molad et al. 2006). In our study, DNA smears were obtained in many of the *A. marginale msp1β* secondary nPCR products from field samples. Smearing in PCR products can indicate the addition of too much template DNA (http://www.bio-rad.com/en-za/applications-technologies/pcr-troubleshooting); however, the amount of primary PCR product added was optimised, and many positive samples gave discrete PCR products, indicating that this was probably not the cause of the smears. Smearing can also result if the sequence of one of the primers does not correspond with the sequence of the template. Cloning and sequencing of the *msp1β* gene of *A. marginale* from selected field samples revealed a 12-bp deletion in the target region of the secondary PCR forward primer (AM100). This primer would almost certainly fail to anneal to *A. marginale* strains containing the deletion, therefore yielding false negative results. The smears obtained in many of the *A. marginale msp1β* secondary PCR products from field samples were therefore likely to be due to the presence of the deletion in the *msp1β* gene in these samples. The use of a forward primer targeting a more conserved region of the gene would overcome this problem.

Although *A. centrale* is considered to be less pathogenic than *A. marginale*, the vaccine strain has been reported to cause severe anaplasmosis in adult cattle of susceptible breeds and in splenectomised adult cattle (Bigalke 1980; Kuttler 1966; Pipano et al. 1976, 1985). More recently, a clinical case of bovine anaplasmosis attributed to a pathogenic strain of *A. centrale* that is closely related to the vaccine strain was reported in Italy (Carelli et al. 2008). It is therefore important to use assays that are specific and sensitive in detecting both *A. marginale* and *A. centrale.* Our results indicate that the qPCR assay is ten times more sensitive than the nPCR assay in detecting *A. centrale* infections. These results are corroborated by the higher prevalence of *A. centrale* detected in field samples by the qPCR assay than the nPCR. However, Decaro et al. (2008) found the nPCR assay to be 1 log more sensitive than the qPCR assay in detecting *A. centrale* infections in cattle. The nPCR targets the multi-copy *msp2* gene and would be expected to be more sensitive than assays that target single-copy genes (Hofmann et al. 2015; Reinbold et al. 2010). However, *msp2* is also a highly variable gene, and assays utilising such genes should target conserved regions of the gene so that the assay detects infections from a wide variety of hosts and geographical regions. Although we were not able to sequence the *msp2* gene of samples with conflicting *A. centrale* nPCR and qPCR results, it is possible that this discrepancy is because of sequence differences in one or more of the primer target regions of South African *A. centrale* strains, as we observed with *A. marginale msp1β* sequences from South Africa. Variation of the *msp2 gene* of *Anaplasma* spp. has previously been shown to occur between and within species, and amongst geographically different isolates (Rymaszewska 2011).

Although probe-based qPCR is more expensive than nPCR, it offers more advantages in that it is usually more sensitive, it is quantitative and has a short turn-around time, and the risk of carry-over contamination is much less than nPCR (Carelli et al. 2007). Additionally, the duplex qPCR assay developed by Decaro et al. (2008) offers a multiplex assay for simultaneous detection of low infections of both *A. marginale* and *A. centrale* using species-specific primers and probes in a single assay. It is therefore an invaluable tool for specific detection of these organisms in endemic regions.

## Conclusion

Our results indicate that the duplex qPCR is more sensitive than the nPCR and RLB assays in detecting carriers of bovine anaplasmosis in South Africa. The RLB is the least sensitive method and detected fewer field samples than could be detected by the other methods. We found that there is variability in the *msp1β* gene target region of one of the internal primers of the nPCR assay. This highlights the importance of testing the suitability of these assays in a new geographical region prior to deployment and also the difficulty of designing tests for these variable pathogens. Surface proteins are often attractive targets as they provide good species specificity; however, these molecules are under tremendous selection pressure and are therefore frequently variable.
